# The Effect on Hemostasis of Gelatin-Graphene Oxide Aerogels Loaded with Grape Skin Proanthocyanidins: In Vitro and In Vivo Evaluation

**DOI:** 10.3390/pharmaceutics14091772

**Published:** 2022-08-25

**Authors:** Jessica Borges-Vilches, Claudio Aguayo, Katherina Fernández

**Affiliations:** 1 Laboratory of Biomaterials, Department of Chemical Engineering, Faculty of Engineering, Universidad de Concepción, Concepción 4030000, Chile; 2 Department of Clinical Biochemistry and Immunology, Faculty of Pharmacy, Universidad de Concepción, Concepción 4030000, Chile

**Keywords:** proanthocyanidins, *Vitis vinifera* grape skin extract, hemostasis, blood cells

## Abstract

Using in vitro and in vivo models, this study investigated the hemostatic potential to control bleeding of both unloaded gelatin-graphene oxide aerogels and the same loaded with proanthocyanidins (PAs) from *Vitis vinifera* grape skin extract. Our results showed that the physicochemical and mechanical properties of the aerogels were not affected by PA inclusion. In vitro studies showed that PA-loaded aerogels increased the surface charge, blood absorption capacity and cell viability compared to unloaded ones. These results are relevant for hemostasis, since a greater accumulation of blood cells on the aerogel surface favors aerogel–blood cell interactions. Although PAs alone were not able to promote hemostasis through extrinsic and intrinsic pathways, their incorporation into aerogels did not affect the in vitro hemostatic activity of these composites. In vivo studies demonstrated that both aerogels had significantly increased hemostatic performance compared to Spongostan^TM^ and gauze sponge, and no noticeable effects of PA alone on the in vivo hemostatic performance of aerogels were observed; this may have been related to its poor diffusion from the aerogel matrix. Thus, PAs have a positive effect on hemostasis when incorporated into aerogels, although further studies should be conducted to elucidate the role of this extract in the different stages of hemostasis.

## 1. Introduction

Hemostasis is a natural and multifaceted response of the body to control bleeding, in which the combined effect of platelets, blood vessels and the coagulation system itself serve to protect the body from severe blood loss [1,2]. In this process, several enzyme and coagulation factors are responsible for the generation of thrombin and subsequent conversion of fibrinogen into fibrin, the activation of platelets and the promotion of other coagulation factors, leading to the formation of blood clots to control bleeding [3]. Numerous recent studies have provided evidence that natural compounds rich in polyphenols have positive effects on vascular risk, systemic blood pressure, platelet activation and thrombin formation, among others [3,4,5].

A large number of natural polyphenolic compounds have been characterized and their bioactivities have been investigated for a wide range of biomedical applications [6,7,8,9]. Among them are the flavonol compounds, which consist of flavan-3-ols monomers and oligomers with a high content of catechin and proanthocyanidins (PAs) in their chemical structures [10,11]. These compounds can be obtained from the grape seeds (GSE) and skins (GSK) of, e.g., *Vitis vinifera* L. cv. País [6,10,12,13]. PAs have shown a high content of catechin and epicatechin [11,14], which act as bioactive compounds in the prevention of various diseases [15]. In addition, numerous biological activities have been associated with PAs due to their antioxidant, cardioprotective, antimicrobial, neuroprotective and anti-inflammatory properties [16,17,18,19], supporting the use of this natural compound for a wide range of biomedical applications [15].

Despite this wide range of therapeutic properties, the use of polyphenolic compounds has been limited in the biomedical area, due to their sensitivity to environmental changes and instability in physiological media [15,20]. One strategy to overcome these drawbacks is to entrap/adsorb these bioactive natural molecules into different matrices, such as aerogels [6,12,13], nanocomposites [21] and nanofibrous materials [15]. Among them, aerogel-based biomaterials could be used as potent carriers of this phytotherapeutic to overcome its deficiencies.

In our previous work, we demonstrated that graphene oxide (GO)-based aerogels crosslinked with polymers such as polyvinyl alcohol (PVA), chitosan (CS) and gelatin (GEL) can be used as drug delivery platforms for the sustained release of PAs [6,12,13,22]. Our studies also suggested that PA-loaded aerogels have potential to be used as hemostatic devices in the control of profuse bleeding due to their functional properties. Among the PA concentrations evaluated, we have previously demonstrated that aerogels loaded with 5% (*w*/*w*) of PAs have a higher blood absorption and clotting capacity compared to those loaded with 10% PAs [12,22]. As such, this aerogel was applied in the present study. However, past investigations did not include study of the role of PA alone in blood clotting. Nor is it possible, from the available literature, to determine the impact of PAs on hemostasis in order to elucidate their hemostatic activity.

Therefore, this study investigates the hemostatic effect, both in vitro and in vivo, of GEL-GO aerogels loaded with PAs from *Vitis vinifera* grape skin extracts. A comparison of the physicochemical and mechanical properties of both aerogels, i.e., with and without PA, was performed to investigate the effect of this bioactive compound on the aerogel properties. The in vitro hemostatic potential of PAs was evaluated by determinations of blood coagulation capacity and clotting activity, as well as through cytotoxicity assays. In addition, a rat-tail amputation model was used to investigate the in vivo hemostatic effect of PA-rich extracts loaded into aerogel matrices, with the results being then compared with those of a commercial hemostat (Spongostan^TM^, Johnson & Johnson, New Brunswick, NJ, USA) and a gauze sponge. To our knowledge, this is the first study to investigate the in vitro and in vivo hemostatic potential of PAs combined with GEL-GO aerogels and to contrast them with commercial materials.

## 2. Materials and Methods

### 2.1. Materials

País grapes, harvested in the Quillón Valley, Bio Bio Region, Chile, were collected and kept in sealed bags at −18°C to minimize polyphenol deterioration. Folin-Ciocalteu reagent, (+)-catechin, (−)-epicatechin and phloroglucinol were purchased from Sigma-Aldrich Company (St. Louis, MO, USA). Sodium carbonate was supplied by Winkler and Zawadzky (Santiago, Chile). All other reagents and solvents used, such as methanol, n-hexane, ethanol, acetic acid, hydrochloric acid, acetonitrile and acetone, were purchased from Merck (Darmstadt, Germany). Tetrahydrofuran (THF) and polystyrene EasiCal standards were supplied by Panreac (Barcelona, Spain) and Agilent Technologies (Madrid, Spain), respectivel

Other reagents, such as dimethyl sulfoxide (DMSO), Dulbecco′s modified Eagles medium (DMEM), fetal bovine serum (FBS) and 3-(4,5-dimethylthiazol-2-yl)-2,5-diphenyltetrazolium bromide (MTT) were purchased from Sigma Aldrich (Saint Louis, MO, USA) and used in in vitro assays. Human dermal fibroblast (HDF) cells were supplied by Sigma-Aldrich Company. The in vitro assays also used fresh human blood, obtained from healthy volunteers, and a standard medical gauze (hydrophilic, 100% cotton, aseptic and sterile), purchased from a commercial pharmacy. Milli-Q^®^ and distilled water were used in this study. All reagents were used as received, without further purification.

### 2.2. PA Extraction from País Grape Skin

PA-rich extracts were obtained from *País* skin grapes following the protocol previously proposed by Morales et al. [23] with some modifications. Briefly, the skins of 200 grapes were manually separated. The extraction was performed in a 500 mL Erlenmeyer flask containing 250 mL of a 2:1 (*v*/*v*) acetone/water solution. This solution was stirred in a rotary shaker (New Brunswick Scientific Co. Edison, NJ, USA) for 15 h at room temperature under dark conditions to prevent oxidation. The acetone content present in the solution was removed using a rotary evaporator (Bibby Sterilin Ltd., RE-100B, STONE Staffordshire, England) operating at reduced pressure and temperature conditions (<35 °C) until 50 mL of GSK was reached. The liposoluble components contained in the GSK were also removed with n-hexane (50 mL) by three washing steps.

### 2.3. Purification of PAs

PA-rich grape extracts were purified in a size exclusion chromatography column using Toyopearl HW-40F resin packed in an Omnifit column (flux rate: 7.0 mL/min, 420 Å∼35 mm). Prior to purification, an ethanol/water solution (55:45 *v*/*v*) was used to equilibrate the column, and the phenolic acids and sugars contained in the extracts were extracted. Next, an acetone/water solution (60:40 *v*/*v*) was used to elute the PA fraction. The acetone content was then removed again using a rotary evaporator working at reduced pressure and temperature conditions (one column volume). Finally, the obtained extracts were freeze-dried (Labconco freeze-dry system, Kansas City, MO, USA) for 72 h and kept at 4 °C for further analysis.

### 2.4. Characterization of PAs

The total phenol content (TPC) of PAs contained in the grape skin was determined by Folin-Ciocalteu assay, as previously described by Jerez et al. [24]. Briefly, 2.5 mL of Folin-Ciocalteu reagent was dissolved using 2.0 mL sodium carbonate solution (7.5% *w*/*v*) and 0.5 mL of the diluted extract. The above solution was heated for 15 min at 45 °C and its absorbance was measured at 765 nm (Spectroquant^®^ Prove 600 spectrometer; Merck KGaA, Darmstadt, Germany). The TPC, expressed as units of milligram equivalent of gallic acid per milligram of extract, was calculated by Equation (1).

(1) $$F_{t}=f_{c}\times A_{bs}\times D_{i}$$

where *F_t_* is the total phenol content (gram equivalent gallic acid per gram of extract), *f_c_* is an equivalence factor (0.0116 L solution/mg equivalent gallic acid) and *A_bs_* and *D_i_* represent the absorbance of the solution (nm) and the sample dissolution, respectively.

To characterize the PA-rich extracts, their degree of polymerization (mDP) and average molecular weight (aMW) were measured. In addition, the phloroglucinolysis method, proposed by Kennedy and Jones [25], was used to determine the molar composition of these extracts. A solution of 0.1 moleq./HCl in methanol containing 50 g/L of phloroglucinol was reacted with skin extracts (5.0 g/L) for 20 min at 50 °C. Then, the final mixture was combined with five volumes of aqueous sodium acetate 40 mM to stop the reaction. The compounds were detected by High Performance Liquid Chromatography (HPLC) at a wavenumber of 280 nm. For this purpose, a Merck-Hitachi chromatograph (LaChrom L7000 Series) with an ultraviolet (UV) detector L-4250, gradient pump L-7100, autosampler L-7200 and two Chromolith Performance Series RP- 18e columns (Merck, Darmstadt, Germany) were used. In these measurements, the following mobile phases were used: mobile phase A, consisting of Milli-Q^®^ water with 1% *v*/*v* aqueous acetic acid; mobile phase B, containing acetonitrile with 1% *v*/*v* acetic acid; and the elution, performed using a 3% B for 4 min. In addition, the following linear gradients were set: 80% B for 2 min (flow rate of 3 mL/min and temperature of 30 °C) and 3–18% B for 14 min. To avoid impurities, the chromatographic column was washed before each injection with 3% B for 2 min. An external catechin standard (100 mg of C/L) was used to quantify the sample. This procedure was performed in duplicate with reproducible results.

Gel permeation chromatography: This assay was carried out following the protocol described by Williams et al. [26]. Briefly, pyridine and acetic anhydride (2.0 mL, 1:1, *v*/*v*) were used to acetylate 50 mg of PA-rich extracts. The reaction was kept overnight at room temperature. Then, the acetylated extracts were dissolved in tetrahydrofuran THF (10–15 mg/mL) and the solvents were removed by evaporation. Then, an HPLC (ACME 9000, Young Lin Instrument Co., Ltd., Anyang, Korea), equipped with two PSS SDV gel columns of 30 cm in length (5 µm, 100 and 500 Å), a PSS SDV gel precolumn (5 µm) equilibrated at 23 °C, and an UV/VIS detector was used to determine the molecular weight distribution of the grape extracts. For the detection of the analytes, the following conditions were used: flow rate of 1.0 mL/min for the mobile phase (THF) and an injection volume of 20 µL. The measurements were performed at a wavenumber of 254 nm. Furthermore, ten polystyrene standards with different molecular weights (Mw 162–19,950 Da) were used.

Finally, the functional groups of these extracts were identified by Attenuated Total Reflection Fourier-Transform Infrared Spectroscopy (ATR-FTIR). The spectra were measured in a wavenumber range between 4000–400 cm^−1^ using a spectral resolution of 4 cm^−1^ and 32 scans.

### 2.5. Loading PA into Aerogels

GEL-GO aerogel matrices were used as carriers to load PAs from grape skin according to the synthesis protocol described in our previous work [12]. Briefly, a GEL-GO solution was previously prepared through a microwave (MW)-assisted reaction under the following synthesis conditions: the pH of the GO solution was 11 and the GEL:GO ratio was 10:1. After this process, 0.055 g of grape skin extracts, equivalent to 5% (*w*/*w*) of the total mass of the aerogel (considering GO and GEL contents), were weighed and dissolved into 20 mL of Milli-Q^®^ water. Thus, the PA concentration was 2.75 mg/mL, which represents the highest theoretical concentration of PAs loaded in the aerogel. The PA solution was slowly added to the GEL-GO solution, and the final mixture was stirred at room temperature until homogenized. To form the aerogels, the mixtures of GEL-GO and PA-loaded GEL-GO were crosslinked for 12 h, frozen at −86 °C and then lyophilized.

### 2.6. Influence of PAs on Aerogel Properties

The effects of loading PA-rich extracts on the physicochemical and mechanical properties of aerogels, i.e., their apparent porosity, elastic modulus and surface charge, were investigated as previously described [12].

Apparent porosity: The apparent porosity of aerogels was measured based on a pycnometer analysis, as previously described [27,28]. Briefly, aerogel samples (1.0 cm^3^) were immersed in a pycnometer containing an ethanol solution (99%, 0.789 g/mL density). During this test, different weights of the pycnometer and solution were measured, as follows: dry weight of the pycnometer (*W_O_*), weight of ethanol and pycnometer (*W_W_*), weight of ethanol, pycnometer and aerogel sample (*W_T_*) and weight of the pycnometer and aerogel sample, both dry (*W_P_*). The actual volume (*V_a_*) was determined according to Equation (2):

(2) $$V_{a}=\frac{\left(\left(W_{W}-W_{O}\right)-\left(W_{T}-W_{P}\right)\right)}{0.789}$$

Then, the apparent porosity was calculated using Equation (3):

(3) $$Apparent\;porosity\;\left(\%\right)=\frac{V_{d}-V_{a}}{V_{a}}\times 100$$

Elastic modulus: The elastic modulus of each aerogel was determined by a uniaxial compression test. The aerogels were compressed up to 480 kN using a universal testing system (Instron Model 4468, Norwood, MA, USA) operating a load capacity of 0.001~500 kN and a loading rate of 1 mm/min. The elastic modulus was determined as the slope of the first linear region (between 10% and 40%) of the strain axis in the stress-strain curves.

ζ-potential measurements. The surface charge of PA-rich extracts and aerogels was measured by the ζ-potential determinations using the Dynamic Light Scattering principle (Nano particle analyzer SZ-100 Horiba Scientific, Kyoto, Japan). Each sample (1.0 cm^3^) was dissolved in a phosphate tampon solution (PBS) under physiological conditions (pH = 7.4, 37 °C) with stirring and subsequently sonicated for 20 min before measurement.

### 2.7. In Vitro Hemostatic Studies

The in vitro hemostatic performance of aerogels loaded with PA-rich extracts was evaluated by comparing their blood absorption capacity, clotting activity and cell viability with those of unloaded aerogels, as described in our previous work [12]. The following assays were used to determine the aforementioned parameters.

Blood absorption capacity: The blood absorption capacity of the two aerogels was evaluated using PBS and a fresh human blood sample, both under physiological conditions. For this purpose, each liquid medium was dropped separately onto each aerogel sample (1.0 cm^3^) until saturation. The liquid supernatants were then removed with filter paper and the absorption capacity was calculated according to Equation (4):

(4) $$Absorption\;capacity\;\left(g/g\right)=\frac{W_{wet}-W_{dry}}{W_{dry}}$$

where *W_wet_* represents the weight of the wet sample after contact with the liquid media) and *W_dry_* is the dry sample weight. Finally, the absorption capacities were expressed as grams of liquid per gram of aerogel (g/g).

Clotting activity: In accordance with previous methodologies described in the literature [12,29,30], the clotting activity of aerogels and PA-rich extracts was determined by measuring the prothrombin time (PT), activated partial thromboplastin time (aPTT) and soluble P-selectin levels. For these measurements, a platelet-poor plasma (PPP) sample was obtained by centrifuging fresh human blood for 10 min at 3000 G. Then, 500 µL of PPP was added to each aerogel sample (1.0 cm^3^), followed by incubation at 37 °C for 30 min. To measure PT, 100 µL of PT reagent was mixed with 200 µL of PPP in a test tube and incubated at 37 °C for 3 min. An automated blood coagulation analyzer was then used to measure the PT (Rayto RT-2204C, Shenzhen, China). The same procedure was used to measure the aPTT, in which 100 µL of PPP with 100 µL of aPTT reagent were incubated at 37 °C for 3 min. Finally, the resulting solution was treated with 100 mL of aqueous calcium chloride (CaCl_2_, 0.03 M), and the aPTT was determined using the same instrument. In parallel to these assays, 500 µL of PPP, also separated from the fresh human blood, were incubated at 37 °C for 30 min (without any test material) and used as a control group in the PT and aPTT measurements.

Plasma soluble P-selectin levels were also measured to assess the activation of platelets adhered to aerogels. Briefly, a fresh human blood sample was centrifuged for 10 min at 2000 G to obtain platelet-rich plasma (PRP). After adding 200 µL of PRP, each aerogel sample was incubated at 37 °C for 30 min under static conditions. Finally, plasma soluble P-selectin levels were quantified with an ELISA kit (Thermo Fisher, Waltham, MA, USA) according to the manufacturer′s instructions.

Cell viability assays: The in vitro cytotoxicity of PA-rich extracts and the aerogels was assessed on human dermal fibroblast (HDF) cells by cell viability (c.v.) determinations. Briefly, 1 mL DMEM was added to 10 mg of each test material, both containing the highest concentration of PAs (2.75 mg/mL). These materials were incubated at 37 °C for 24 h to recover the supernatants. The final PA concentrations released to the cell medium were also determined. In the case of the PA-loaded aerogel, the concentration of PA released was determined based on the results of our previous release studies [12], in which about 7% of the total PA content was released to the medium after 24 h of assay, which is equivalent to a final PA concentration in the cell medium of 0.2 mg/mL. In the case of pure PA-rich extracts, there was no change in the PA concentration and, therefore, the final PA concentration released to the medium was 2.75 mg/mL. The recovered supernatants were then combined with 2% (*v*/*v*) FBS, 1% (*v*/*v*) antibiotics (100 units/mL of penicillin and 100 units/mL of streptomycin) and 1% amphotericin to serve as complete medium and prevent fungal proliferation in the cells. After that, the supernatants were poured over HDFs (10^4^ cells/mL) in 96-well plates and incubated for 48 h at 37 °C with humidified air with 5% (*v*/*v*) CO_2_. The HDF cells were then rinsed with PBS (pH = 7.4, 37 °C), and the supernatant was drained. Next, fresh DMEM (100 µL) were added to the HDF cells and a test kit containing 5 mg/mL of MTT was utilized to measure cell viability. For this, HDF cells were cultured for 4 h at 37 °C with CO_2_, then 85 mL of medium was removed, and 50 mL of DMSO was added. Equation (5) was used to calculate cell viability, as follows:

(5) c.v. (%)=(Abs540 testAbs540 control)×100

where *A_bs_* represents the absorbance value, measured at 540 nm by UV-VIS spectrometry (Spectroquant^®^ Prove 600 spectrometer, Merck KGaA, Germany). HDF cells without material were used as a control in this assay.

### 2.8. In Vivo Hemostatic Assays

The in vivo hemostatic potential of PA-loaded aerogels was investigated through a comparison between unloaded and loaded aerogels using a rat-tail amputation model. The hemostatic time and amount of blood loss of each tested material were determined according to methodologies described previously [31,32,33,34] with slight modifications. Healthy Sprague Dawley (SD) rats, weighing 262 ± 12 g and aged 14 weeks, were received and treated according to the protocols established by the Animal Ethics Committee of Universidad de Concepción for the care and use of laboratory animals. In order to ensure a healthy physiological condition, the rats were initially housed in cages and quarantined under a standard environment. For this assay, aerogel samples were synthesized in the shape and dimensions of a 12-well plate, while Spongostan^TM^ (commercial material) and a gauze sponge, both cut to the same size of aerogels, served as controls. Prior to this assay, each sample was sterilized for 1 h under UV radiation. Before surgery, the rats were anesthetized with a ketamine/xylazine solution and divided into four groups of five animals each. After that, a sharp knife was used to cut the rat tail at its midpoint. To ensure normal blood flow, the rat tail was placed in air for 15 s after cutting. Afterwards, each test material was immediately placed in contact with the wound section and slight pressure was applied to control bleeding. During this test, we recorded hemostatic time based on the criteria that no active bleeding or visible blood was present in the wound section. In addition, blood loss was determined by the difference in weight of the material before and after the test.

### 2.9. Statistical Analysis

Each experiment was carried out three times and the measurements were performed in quadruplicate with reproducible results. Data analyses were performed using OriginPro8.5^®^ software (Northampton, MA, USA). All data comparisons were made using analysis of variances (ANOVA) with an accepted significance of *p*-value < 0.05 and the multiple range analysis (Duncan′s test, 95% confidence) were performed with Statgraphics Centurion XVII^®^ software (NIH, Bethesda, MD, USA). The results are shown as the mean ± SD value, and the error bars are included in each figure.

## 3. Results and Discussion

### 3.1. Characterization of PAs

**Figure 1:**
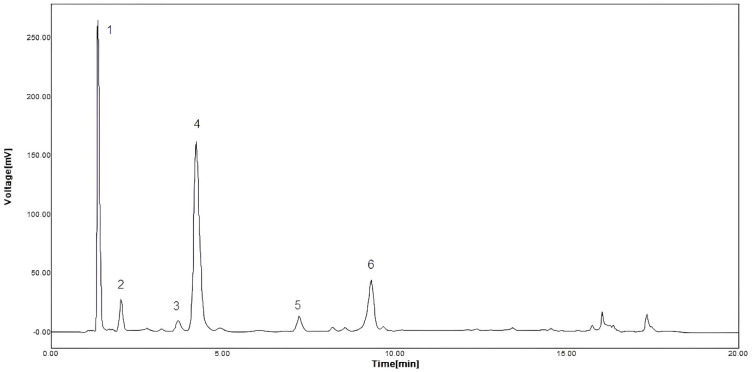
HPLC chromatogram of the grape skin extract after acid-catalyzed depolymerization. The main compounds identified are: (1) phloroglucinol, (2) EGC-P: (─) epigallocatechin- phloroglucinol, (3) C-P: (+)-catechin-phloroglucinol, (4) EC-P: (─)-epicatechin-phloroglucinol, (5) C: (+)-catechin, and (6) ECG-P: (−)-epicatechin gallate phloroglucinol [11]. Reprinted from LWT-Food Science and Technology, 44, Inhibition of the angiotensin-converting enzyme by grape seed and skin proanthocyanidins extracted from *Vitis vinífera* L. cv. País, Authors: Gonzalo Eriz, Verónica Sanhueza, Marlene Roeckel, Katherina Fernández with permission from Elsevier (2011).

The PAs contained in GSK were characterized for their TPC, aMW, mDP and type of flavan-3-ols subunits. The TPC, aMW and mDP values obtained for these extracts were: 0.6 ± 0.2 g equivalent gallic acid/g extract, for 1 g/L of solution, 7565 ± 221 (molecular weight units) and 25.5 ± 0.8 (dimensionless), respectively. In addition, seven different phenolic compounds were identified (Table 1), with a prevalence of epicatechin gallate-phloroglucinol (EC-P) and epigallocatechin-phloroglucinol (EGC-P) in the structural composition of these extracts. These results were also corroborated by the HPLC chromatogram obtained for the grape skin extracts, as shown in Figure 1. Five different compounds, similar to those mentioned above, were detected in this chromatogram, with a prevalence of EC-P as the major component in this type of grape skin extract. In addition, the compounds epigallocatechin (ECG) and (−)-epicatechin (EC) were only identified as extension subunits in these extracts.

**Figure 2:**
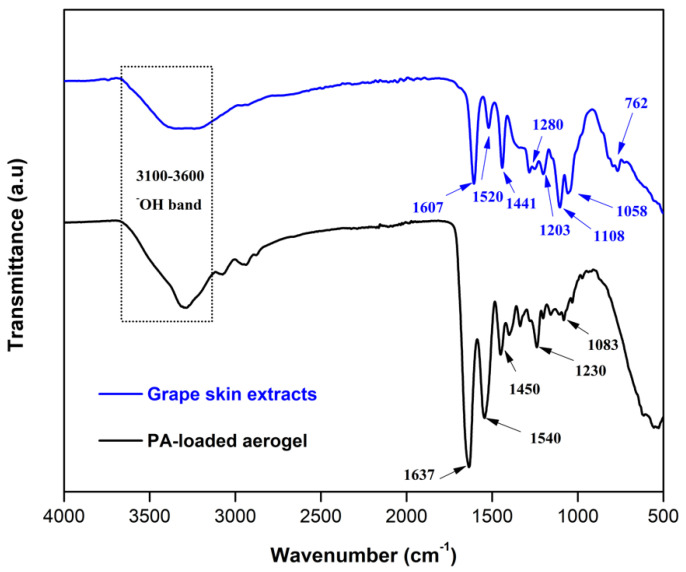
ATR-FTIR spectra of grape skin extract and PA-loaded aerogel showing their main functional groups.

As listed in Table 2, the molecular weight distribution of these extracts was between 451 and 25,807 g/mol. Furthermore, their functional groups were identified by ATR-FTIR (Figure 2), showing characteristic bands of these extracts, such as the aromatic C-H extensions of aromatic rings in-plane and out-of-plane bend at wavenumbers of 950–1200 cm^−1^ and 700–880 cm^−1^, respectively. In addition, we identified C=C-C aromatic ring stretching between 1400–1520 cm^−1^ and 1604–1612 cm^−1^, a C-O stretch band belonging to the pyran-derived ring structure between 1200–1280 cm^−1^ and hydroxyl groups (-OH) in the range of 3200–3400 cm^−1^ [35]. These results provide information on the chemical characteristics and functional groups of PAs, suggesting that these extracts are large and complex molecules with beneficial functional attributes for coagulation. A comparison of the structural characteristics of PAs from *País* grape extracts with those reported for PAs from other grape cultivars, determined by a depolymerization method, showed differences in their terminal subunits compared to the Cabernet Sauvignon and Merlot varieties due to the absence of (−)-epicatechin (EC) [14]. However, in their chromatograms, other authors have identified a similar structural composition for PAs from grape skin [10].

To investigate the physicochemical interactions between the PAs and the GEL-GO aerogel once incorporated, the ATR-FTIR spectrum of the PA-loaded aerogel was analyzed (Figure 2). In this spectrum, the presence of the characteristic functional groups of GEL and GO was observed, such as an alkoxy stretching at 1048 cm^−1^ belonging to the GO structure. In addition, vibrations of amide I, amide II and amide III of the GEL chains were identified at 1637 cm^−1^, 1540 cm^−1^ and 1230 cm^−1^, respectively. We also found a peak at 1450 cm^−1^, attributable to the stretching vibrations in the pyrrolic ring of the GEL chains. However, it was not possible to identify new peaks associated with the PA in the spectrum of the PA-loaded aerogel. This may have been due to the limited PA concentration loaded in the composite compared to the GO and GEL contents, leading to a predominance of bands of GEL and GO in the ATR-FTIR of the aerogel. These results suggest that the PAs were non-covalently bound to the GEL-GO matrices, which has been previously demonstrated [12]. In this regard, phenolic compounds have the ability to act as hydrogen-bond donors and may react through hydrophobic interactions, involving the planar aromatics of polyphenols and the hydrophobic sites of substrate molecules, such as GEL [36].

### 3.2. Influence of PA Loading on the Aerogel Properties

The effects of loading PAs into aerogels were investigated in relation to the physical and mechanical properties of these composites (Table 3). Both aerogels presented apparent porosities higher than 90% and elastic moduli in the order of 2.5 to 2.8 kPa; these values were similar to those reported by other authors for hydrogels and aerogels composed of GEL and GO for biomedical uses [37,38]. The addition of these extracts did not affect any of the evaluated properties. On the contrary, the incorporation of PAs into aerogels increased their negative surface charge density by 34% compared to unloaded aerogels. The PA-rich extracts had a surface charge around –31 mV; this negative character was due to the presence of oxygenated functional groups in the chemical structure of the extracts. Although there was a strong decrease in the surface charge of the aerogels compared to the PAs alone, both maintained a negative character, which is favorable to interfacial stimulation with blood cells [6,34]. These results suggest that the incorporation of PA into aerogels could help hemostasis due to a charging effect of the material in its interaction with blood cell components, as well as its high porosity.

In hemostatic applications, the modification of the physicochemical and structural properties of materials due to the incorporation of new molecules is essential. These changes make it possible to improve current hemostatic materials or to induce new hemostatic properties into materials, regardless of their hemostatic background [39]. One of the most widely used strategies to improve material–blood interactions has been interfacial stimulation on the material surfaces [32,33,34]. Surface charges, both negative and positive, play a key role in the interaction of the material with blood coagulation processes. Negative charges are beneficial for the adsorption of platelets and red blood cells (RBCs) on the surface materials, while positively charged surfaces can interact with plasma and increase plasma coagulation through the intrinsic coagulation pathway [40,41]. In this study, we postulate that the incorporation of PA into aerogels could improve aerogel–blood cell interactions due to a charging effect, which will be discussed later.

### 3.3. In Vitro Hemostatic Potential of PAs

The absorption capacity of materials is an essential property for wound dressing applications, since it determines the liquid volume absorbed by the material. PA-loaded aerogels showed a higher blood coagulation capacity compared to unloaded ones, with values of 48% and 18%, respectively. This strong increase could be mainly attributed to the chemical composition of the grape extracts, which contained abundant phenolic groups, allowing them to act as donors in hydrogen bonding to interact with blood cells. Similarly, these extracts had a high content of oxygenated functional groups, which could instantly stimulate RBCs and platelets on the aerogel surface through electrostatic interactions [19]. Both interactions are beneficial for hemostasis, since an increase in the amount of blood absorbed by aerogels could increase the number of blood cells accumulated on their surfaces, thus favoring blood clot formation and accelerating hemostasis.

The in vitro hemostatic potential of the PA-rich extracts was further investigated by aPTT and PT determinations (Table 4). Comparatively, both clotting parameters were lengthened in the presence of the PA compared to control plasma, indicating that these extracts alone do not promote coagulation through intrinsic and extrinsic pathways. Although grape extracts have shown interesting therapeutic properties for a wide range of biomedical applications, their role in blood clotting remains unclear. The multifactorial nature of the coagulation process could explain those controversial results. Blood clotting is a process is influenced by several factors, such as the physicochemical nature and the surface properties of the materials [42,43]. PAs from grape skin have a high content of oxygenated functional groups, which provides a negative surface charge density, and a predominance of hydrophilic chains that give them a hydrophilic nature. Both characteristics are favorable to the interfacial stimulation of platelets and RBCs on the surfaces of the materials when they come into contact with blood cells [34,44].

The clotting activity of both aerogels was also measured to investigate the role of PAs in the blood clotting process (Table 4). When the aerogels came into contact with blood, no coagulation effects were triggered through the extrinsic and intrinsic pathways, as evidenced by increased aPTT and PT values compared to the plasma control (*p*-value < 0.05). In addition, P-selectin levels decreased significantly in both aerogels compared to the control, with this reduction being more pronounced in the PA-loaded aerogels. Our results suggest an inhibitory effect of PAs on the blood coagulation process, which could be associated with the ability of these compounds to interact with platelets, phospholipids and proteins [45,46]. In a related study, Sinegre et al. [4], who investigated the role of epicatechin (a major flavonoid compound in grape extracts) in blood coagulation, demonstrated that the inhibitory capacity of this compound in the enzymatic reactions of the coagulation cascade is due to its low impact on PT and aPTT. Furthermore, previous studies have shown that negatively charged materials can interact with blood cells to promote hemostasis through alternative coagulation pathways [47], which is relevant for hydrophilic materials containing hydroxyl and amine groups in their structures which are capable of covalently binding to complement protein C3b [48]. Therefore, we expect that the inclusion of PAs will improve the hemostatic performance of aerogels due to the incorporation of new functionalities into their matrices.

Although PT and aPTT assays are considered conventional methods for assessing plasma coagulation, they have revealed certain shortcomings that make it difficult to extrapolate their results to in vivo models [4]. These assays do not take into account all the factors involved in plasma coagulation, in particular, anticoagulant factors. Similarly, only the first traces of thrombin, but not the thrombin burst phase, are considered [49]. Therefore, further studies, such as thrombin generation assays, are needed to assess the overall impact of PA on hemostasis. In addition, the use of a positive control group in these assays is also suggested to contrast the obtained results.

**Figure 3:**
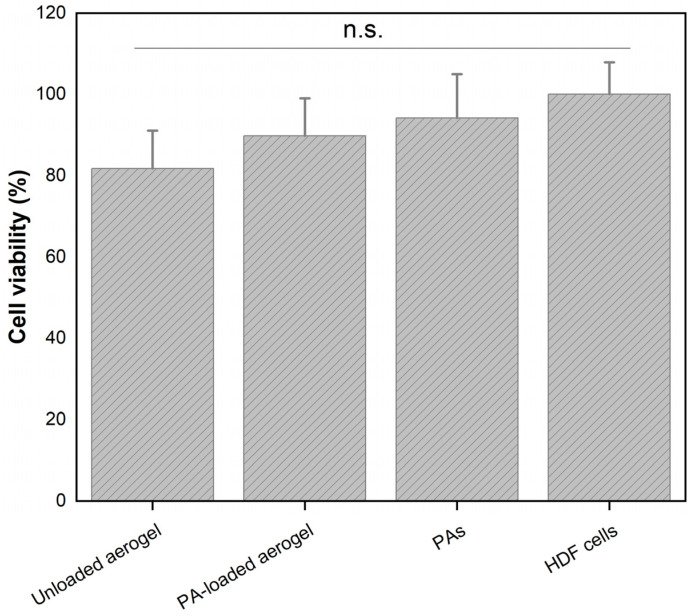
In vitro cell viability values for PAs and aerogels evaluated on HDF cells. Data correspond to mean ± SD value (*n* = 6, n.s.: non-significant statistical differences, *p*-value < 0.05).

The in vitro cytotoxicity of PA-rich extracts and the aerogels was assessed by cell viability assays in HDF cells. As explained in Section 2.7, both materials were initially prepared at the highest PA concentration, i.e., 2.75 mg/mL, and after incubation, the PA content released from the aerogel to the cell medium was 0.2 mg/mL. Figure 3 shows that grape skin PAs alone had a cell viability of around 94%, indicating their non-toxic effects at this study concentration. This result may be associated with the chemical composition of grape skin, which has shown favorable effects on fibroblast activity [12,13,15]. When comparing the aerogels, we observed that the PA-loaded aerogel showed a slight increase in the cell viability values compared to the unloaded aerogel, with values close to 90% and 82%, respectively. These results can be attributed to the beneficial effects of PAs on fibroblast cells, and are in agreement with previous studies [7,45,50,51,52]. Thus, these findings demonstrate that the inclusion of PAs improves the in vitro biocompatibility of these aerogels, which is favorable for hemostasis, as it promotes cell migration to the wound section.

### 3.4. In Vivo Hemostatic Potential of PAs

**Figure 4:**
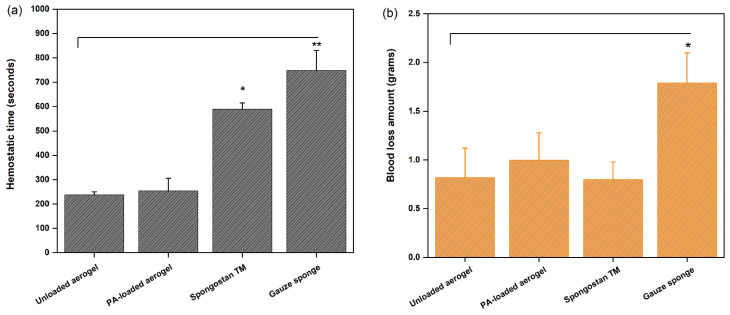
(**a**) Hemostatic times and (**b**) blood loss amounts, both measured during the in vivo assay for PA-loaded aerogel, unloaded aerogel, Spongostan^TM^ and control gauze sponge. * *p* ≤ 0.05, ** *p* ≤ 0.01.

A rat-tail cutting experiment was employed to investigate the in vivo hemostatic potential of PA-loaded aerogels, with their in vivo hemostatic efficacy being compared with that of an unloaded aerogel. In this assay, the values of hemostatic time and the amount of blood loss were recorded for each test material, including a gauze sponge (control group) and Spongostan^TM^ (commercial material). Both aerogels shortened the hemostatic time by more than 50% compared to other test materials (Figure 4a). This result could be attributed to the rapid formation of blood clots in the rat-tail wound when the aerogels came into contact with the injury site. Blood clot formation is one of the most important processes involved in the blood coagulation cascade, as it promotes the accumulation of blood cells on the surface of the materials to accelerate hemostasis. In addition, both aerogels reduced the amounts of blood loss by 44% compared to the control gauze (Figure 4b), while the blood loss of Spongostan^TM^ was comparable to that of both aerogels, possibly due to the high GEL content incorporated into these materials. The inclusion of PAs had no significant influence on any of the evaluated parameters, which could be due to the limited concentration of PAs loaded into the aerogel. These results confirm, on the one side, that the gauze itself only has a positive effect in the control of bleeding, but not in the promotion of hemostasis. On the other side, both aerogels achieved faster hemostasis than Spongostan^TM^ due to a significantly reduced hemostatic time, suggesting the superior hemostatic efficacy of our aerogels in controlling profuse bleeding.

Our results showed that the unloaded and loaded aerogels presented similar hemostatic behavior in vivo, which was not replicated from our previous in vitro clotting results, demonstrating that the PA-loaded aerogel had a greater of blood absorption capacity and amount of coagulated blood compared to the unloaded aerogel [12]. The observed differences could be attributed to the mode and type of blood contact with the aerogel. In the in vitro assay, whole human blood (500 µL) was directly added to the aerogel samples, and the hemoglobin contents in the supernatant were measured after different reaction times. In contrast, in the in vivo assay, different pulses of tail blood were dropped onto the aerogel samples until coagulation was achieved, reaching a higher mass of retained blood (Figure 4b), which, due to their different diffusion profiles, could dilute the PA amount, thereby reducing the clotting effect observed above. Therefore, further studies using different PA concentrations should be performed to verify the effect of PA dose on coagulation in vivo.

Previous studies have also investigated the in vivo behavior of grape extracts for many biomedical applications. Ozkan et al. [53] investigated the in vivo potential of grape seed proanthocyanidin extracts in the prevention of contrast-induced nephropathy, demonstrating that such natural compounds provide biochemical and histopathological improvement for this disease. Zhao et al. [54] also evaluated the protective effects of GSE against colonic injuries using Wistar rats as an in vivo model. Their study demonstrated that GSE can be used as an effective dietary supplement to prevent such lesions in vivo. Also, other authors have investigated the in vivo potential effects of PA-rich extracts in protecting against different injuries, such as neuronal loss [55] and amiodarone-induced kidney injuries in rats [56]. Soltani et al. [57] also demonstrated that green tea extract-which is a natural compound with high PA content-was significantly effective in stopping bleeding in vivo in the socket created by tooth extraction and in reducing the subsequent oozing. Similarly, Kalalinia et al. [58] showed that the inclusion of green tea in a topical green tea/PVA formulation significantly shortened the hemostatic times in the in vivo rat-tail assay compared to the materials without extracts, thus demonstrating the great potential of these extracts for anti-hemorrhage applications. All these studies confirm the great therapeutic potential of natural extracts in bleeding prevention.

## 4. Conclusions

This study investigated the in vitro and in vivo hemostatic potential of unloaded aerogels and aerogels loaded with PA-rich extracts from grape skin extracts. The addition of PAs did not affect the porosity or mechanical resistance of the aerogels. PA-loaded aerogels showed a higher blood absorption capacity and increased surface charge and cell viability in vitro compared to unloaded aerogels. These results were associated with the increased content of oxygenated groups and oxidation levels after loading PAs into the aerogels, suggesting a beneficial potential of PAs for blood clotting. Based on coagulation activity measurements in vitro, PAs alone showed no noticeable effects on the promotion of hemostasis through intrinsic and extrinsic pathways; however, their inclusion into aerogel matrices favored the in vitro hemostatic activity of these composites. Both aerogels showed similar hemostatic behavior in vivo, and no effects of PA addition alone to the aerogels were observed, probably due to the difference in the extract diffusion from the aerogel matrix. Our findings suggest that the incorporation of PA-rich grape extracts into GEL-GO aerogels could modify the functionalities of these composites to improve hemostasis, thus demonstrating a novel potential application for this phytotherapeutic compound. However, further studies using different PA concentrations should be conducted to better elucidate the role of these extracts as hemostatic agents in vivo.

## Figures and Tables

**Table 1:** Structural composition of grape skin extracts.

Compounds	Content (µM)
(+)-catechin (C)	156 ± 22
(−)-epicatechin (EC)	not detected
epigallocatechin (ECG)	not detected
(+)-catechin-phloroglucinol (C-P)	92 ± 5
(−)-epicatechin-phloroglucinol (EC-P)	2900 ± 152
epicatechin gallate-phloroglucinol (ECG-P)	267 ± 15
epigallocatechin-phloroglucinol (EGC-P)	574 ± 46

**Table 2:** Molecular weight distribution of grape skin extracts.

Molecules Percentage (%)	Molecular Weight (g/mol)
10.5	25,807–14,149
22.7	14,149–8866
55.9	8866–1582
3.8	1582–1156
3.9	1156–791
3.1	791–451

**Table 3:** Physicochemical and mechanical properties of aerogels.

Aerogels	Apparent Porosity (%)	Elastic Modulus (kPa)	Surface Charge (mV)	Blood Coagulation (%)
Unloaded aerogel	91.8 ± 1.4 ^a^	2.5 ± 0.6 ^a^	−3.9 ± 1.1 ^a^	18 ± 2.6 ^a^
PA-loaded aerogel	91.3 ± 1.2 ^a^	2.8 ± 0.3 ^a^	−5.9 ± 1.2 ^a^	48 ± 3.4 ^b^

The letters ^a^ and ^b^ indicate significant statistical differences between samples for *p*-value < 0.05.

**Table 4:** aPTT and PT measurements for PA-rich extracts, aerogels and control plasma.

Samples	aPTT (s)	PT (s)	P-Selectin Levels (ng/mL)
Unloaded aerogel	33.3 ± 0.8 ^a^	13.2 ± 0.8 ^a^	124.6 ± 3.2 ^a^
PA-loaded aerogel	33.9 ± 0.4 ^a^	13.1 ± 0.2 ^a^	92.4 ± 1.9 ^b^
PA from grape skin	40.2 ± 0.8 ^b^	13.1 ± 0.6 ^a^	-
Control plasma	31 ± 0.5 ^c^	11.9 ± 0.5 ^b^	216.5 ± 2.0 ^c^

The letters ^a^, ^b^ and ^c^ indicate significant statistical differences between samples for *p*-value < 0.05.

## Data Availability

Not applicable.
